# RNA interference-mediated knockdown of CD49e (α5 integrin chain) in human thymic epithelial cells modulates the expression of multiple genes and decreases thymocyte adhesion

**DOI:** 10.1186/1471-2164-11-S5-S2

**Published:** 2010-12-22

**Authors:** Leandra Linhares-Lacerda, Marcelo Ribeiro-Alves, Ana Cristina Martins de Almeida Nogueira, Daniella Areas Mendes-da-Cruz, Danielle Aparecida Magalhães, Mireille Dardenne, Geraldo Aleixo Passos, Wilson Savino

**Affiliations:** 1 Laboratory on Thymus Research, Oswaldo Cruz Institute, Oswaldo Cruz Foundation, Rio de Janeiro, RJ, 21040-360, Brazil; 2 Center for Technology Development in Health, Oswaldo Cruz Foundation, Rio de Janeiro, RJ, 21040-360, Brazil; 3 Department of Immunology, National Institute of Quality Control in Health, Oswaldo Cruz Foundation, Rio de Janeiro, RJ, 21040-360, Brazil; 4 Hôpital Necker, Université Paris Descartes, Centre National de la Recherche Scientifique, Unité Mixte de Recherche 8147, Paris, 75743, France; 5 Molecular Immunogenetics Group, Department of Genetics, University of São Paulo School of Medicine, Ribeirão Preto, SP, 14049-900, Brazil

## Abstract

**Background:**

The thymus is a central lymphoid organ, in which bone marrow-derived T cell precursors undergo a complex process of maturation. Developing thymocytes interact with thymic microenvironment in a defined spatial order. A component of thymic microenvironment, the thymic epithelial cells, is crucial for the maturation of T-lymphocytes through cell-cell contact, cell matrix interactions and secretory of cytokines/chemokines. There is evidence that extracellular matrix molecules play a fundamental role in guiding differentiating thymocytes in both cortical and medullary regions of the thymic lobules. The interaction between the integrin α5β1 (CD49e/CD29; VLA-5) and fibronectin is relevant for thymocyte adhesion and migration within the thymic tissue. Our previous results have shown that adhesion of thymocytes to cultured TEC line is enhanced in the presence of fibronectin, and can be blocked with anti-VLA-5 antibody.

**Results:**

Herein, we studied the role of CD49e expressed by the human thymic epithelium. For this purpose we knocked down the CD49e by means of RNA interference. This procedure resulted in the modulation of more than 100 genes, some of them coding for other proteins also involved in adhesion of thymocytes; others related to signaling pathways triggered after integrin activation, or even involved in the control of F-actin stress fiber formation. Functionally, we demonstrated that disruption of VLA-5 in human TEC by CD49e-siRNA-induced gene knockdown decreased the ability of TEC to promote thymocyte adhesion. Such a decrease comprised all CD4/CD8-defined thymocyte subsets.

**Conclusion:**

Conceptually, our findings unravel the complexity of gene regulation, as regards key genes involved in the heterocellular cell adhesion between developing thymocytes and the major component of the thymic microenvironment, an interaction that is a mandatory event for proper intrathymic T cell differentiation.

## Background

The thymus is a central lymphoid organ, in which bone marrow-derived T cell precursors undergo a complex process of maturation, eventually leading to the migration of positively selected thymocytes to the T-dependent areas of peripheral lymphoid organs. This differentiation process involves sequential expression of a variety of membrane proteins and rearrangements in T-cell receptor genes. Most potentially self-reactive thymocytes are negatively selected by clonal deletion, whereas some are rescued from death through positive selection, eventually yielding the vast majority of the T-cell repertoire [1]. In both positive and negative selection events, cell-cell adhesion between developing thymocytes and non-lymphoid microenvironmental cells of the organ is mandatory [1].

The thymic microenvironment which is tridimensional network composed of non-lymphoid cells including thymic epithelial cells (TEC) – the most conspicuous cellular elements – dendritic cells, macrophages and, to a lesser extent fibroblasts, as well as extracellular matrix (ECM) [2,3]. Developing thymocytes interact with thymic microenvironment in a defined spatial order. This is evident from the different localization of the individual stages of thymocyte development. The most immature, CD4^-^CD8^-^ double negative (DN) thymocytes are found beneath the subcapsular epithelium of the thymic lobules, while the more mature, CD4^+^CD8^+^ double positive (DP) stages can be detected throughout the cortical region. The more differentiated CD4^+^ or CD8^+^ single positive (SP) thymocytes are mainly found in the medulla [2]. There is evidence that extracellular matrix (ECM) molecules play a fundamental role in localizing the different thymocyte stages in the thymus [4]. Interestingly, it is possible that supramolecular ECM arrangements function as a conveyor belt, allowing an ordered migration of thymocytes within the organ [5].

Fibronectin (FN) is one ECM ligand constitutively expressed in all mammalian thymuses so far analyzed, including mice and humans [6-8]. Particularly in the human thymus both FN isoforms were detected within the thymic lobules [9]. Two integrin-type fibronectin specific receptors are consistently expressed by developing thymocytes: VLA-5 (α5β1, CD49e/CD29), which recognizes the RGD-containing fibronectin binding site and VLA-4 (α4β1, CD49d/CD29) that identify the CS1 segment of fibronectin, derived by alternative splicing of the corresponding pre-mRNA [10]. VLA-4 is expressed on almost all human thymocytes (98%). Its membrane expression is higher on DN thymocytes, decreases slightly on DP cells, and is 10-fold lower on SP cells. Although VLA-5 is also expressed by the majority of thymocytes (60 to 70%), its expression pattern is distinct from that α4β1: it is expressed at high levels on DN cells, decreases on DP cells, but it is up-regulated on mature single positive cells [11].

Thymic microenvironmental cells express integrin-type fibronectin receptors [4]. In cultured human TEC, both VLA-4 and VLA-5 were detected [12,13]. In the mouse model, we demonstrated that both epithelial and non-epithelial phagocytic cells of the thymic microenvironment express VLA-5 at high densities on their membranes [4,14]. In all cases, the interaction between α5β1 and FN is essential for thymocyte adhesion to microenvironmental cells, since heterocellular adhesion could be significantly disrupted by using anti-VLA-5 antibody applied to growing TEC prior to co-culturing with thymocytes [13-15].

Herein, we further studied the role of integrin α5 subunit in human thymocyte and thymic epithelial cell interactions, through the silencing of the gene coding for CD49e, α5 integrin subunit of VLA-5, in thymic epithelial cells by RNA interference (RNAi), an approach that has emerged as a convenient and effective tool for loss-of-function studies. Using this strategy, we looked for differential gene expression in silenced cells using cDNA microarrays. CD49e gene silencing yielded a modulation of more than 100 genes, including adhesion molecules and some genes related to integrin pathway. Following transfection of cultured TEC with CD49e specific RNAi, we also found that the reduction in VLA-5 density on TEC membrane significantly decreased thymocyte adhesion. Conjointly, our data reinforce the role of VLA-5 in TEC/thymocyte interactions in the process of human thymocyte differentiation.

## Results

### Inhibition of CD49e expression by siRNA in human thymic epithelial cells

**Figure 1:**
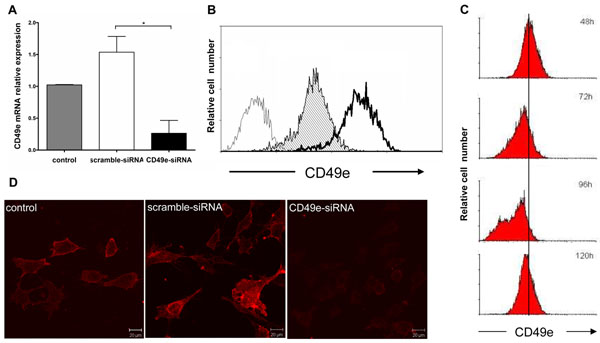
**Knockdown of CD49e gene in human thymic epithelial cells by RNA interference.** Panel A depicts the expression of CD49e mRNA determined by realtime quantitative PCR 48 hours after CD49e-siRNA or scramble-siRNA transfection. Results were normalized to β-glucuronidase mRNA and are expressed relative to the mRNA levels in untreated (non-transfected) cells. Data are representative of three independent experiments performed in triplicate. Bars represent the mean ± SEM; the differences between groups were validated using the Student’s *t* test (p < 0.009). Panel **B** shows flow cytometry profiles of human TEC cultures, for immunochemical detection of CD49e using a specific anti-CD49e monoclonal antibody, 72 hours post-transfection with either scramble-siRNA (gray curve) or CD49e-siRNA (white curve with black contour). The open curve with gray line on the left corresponds to the fluorescence levels elicited by an unrelated isotype matched monoclonal antibody. These plots are representative of four independent experiments. In panel **C**, a kinetic flow cytometry analysis reveals the transient decrease on the membrane expression of CD49e, in cultured human, TEC 48, 72, 96 or 120 hours post-transfection with CD49e-siRNA. Panel **D** reveals immunofluorescence images for detection of CD49e (using the anti-CD49e monoclonal antibody) in human TEC cultures, 72 hours post-transfection with scramble-siRNA or CD49e-siRNA. On the left side of the panel, an untreated TEC culture was submitted to the same immunofluorescence assay. Bars = 20 µm.

We first defined if we could effectively silence CD49e mRNA and the corresponding protein using small interference RNA (siRNA) in human TEC preparations, known to constitutively express α5β1 as well as α4β1 fibronectin receptors [12]. For that, we transfected into the human TEC line a CD49e-siRNA that is complementary to the CD49e gene or a control scrambled siRNA, both conjugated to alexa-488 (applied herein for evaluating the efficacy of transfection). As assessed after 24 hours of transfection in a dose curve by flow cytometry, TECs exhibited 80% of alexa-488 expression compared to labeling seen in untransfected cells in 2.5nM of siRNA (data not shown). The down-regulation of CD49e mRNA induced by siRNA was determined using real-time quantitative PCR. The total level of CD49e mRNA could be reduced as much as 100% on CD49e-siRNA compared to control-siRNA cells, 48 hours following transfection (Figure 1A), although the reduction degree could vary from experiment to experiment, in a range between 40 to 100% of mRNA silencing. To determine whether expression of the CD49e integrin subunit was suppressed on the cell surface of TEC line following transfection with CD49e-siRNA cells were stained with a monoclonal CD49e-specific antibody conjugated to phycoeritrin. A distinct subpopulation with reduced levels of surface CD49e was clearly resolved using flow cytometry in the CD49e-siRNA sample but not in the control sample (Figure 1B). The same result was found by immunofluorescence, transfected cells were stained with the same CD49e-antibody used in flow cytometry 72 hours following transfection. CD49e-siRNA treated cells showed reduction of α5 integrin subunit compared to control siRNA transfected cells (Figure 1D).

A time-course evaluation revealed that CD49e-siRNA-triggered down-regulation of membrane CD49e was transient with most efficient silencing at 72h post-transfection (Figure 1C). Reduced membrane levels of CD49e were not detected earlier than 48h indicating that CD49e specific siRNA treatment requires days to effectively suppress protein expression.

### Specificity of siRNA-mediated silencing

**Figure 2:**
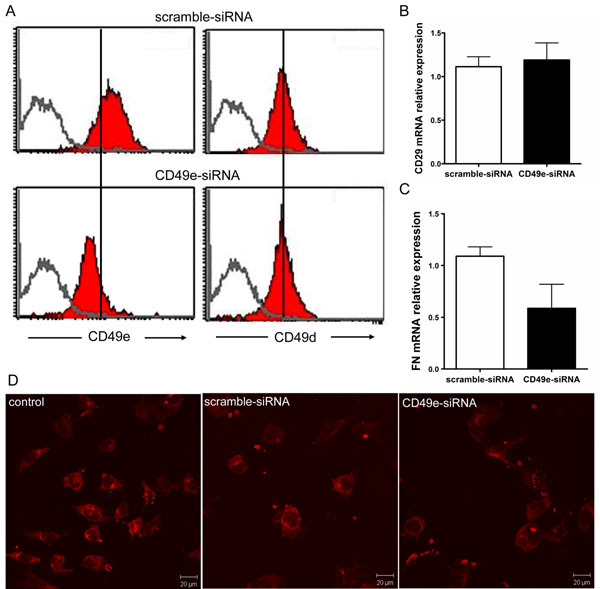
**Specificity of CD49e gene silencing using RNA interference in cultured human thymic epithelial cells.** Panel **A** shows cytofluorometric profiles for membrane detection of CD49e (left panels) and CD49d (α4 integrin chain, right panels) subunits of fibronectin receptors in control, scramble-siRNA and CD49e-siRNA transfected cultured human thymic epithelial cells. There is no change for CD49d expression (red line), although the expression of CD49e is clearly –down-regulated in CD49e-siRNA transfected cells (red line). The vertical black lines were inserted so that to make clearer that membrane levels of CD49e (but not CD49d) were decreased in CD49e-siRNA transfected TEC. Open peaks show the fluorescence signal generated when cells were subjected to the unrelated isotype-matched monoclonal antibody. Panel **B** reveals that there are no changes in the expression of CD29 (β1 integrin subunit) mRNA, as determined by real-time quantitative PCR 48 hours after transfection. Results were normalized to β-glucuronidase mRNA and are expressed relative to the mRNA levels seen in scramble-siRNA transfected cells. Data seen in this panel are representative of three independent experiments performed in triplicate. Panels **C** and **D** show the expression of fibronectin at the mRNA and protein levels respectively. Although the mean levels of mRNA (defined by qPCR) was lower in CD49e-siRNA versus scramble-siRNA transfected cultured TEC, such different was not statistically significant, and the levels of the corresponding protein, herein defined by immunohistochemistry, were similar in both groups (**D**). Panel **D** reveals immunofluorescence images for detection of fibronectin (using the anti-fibronectin monoclonal antibody) in human TEC cultures, 72 hours post-transfection with scramble-siRNA or CD49e-siRNA. On the left side of the panel, an untreated TEC culture was submitted to the same immunofluorescence assay. Bars = 20 μm.

We next investigated whether the surface expression of other integrin-type fibronectin receptor was altered in CD49e-siRNA transfected cells. For that, we analyzed the cell surface expression of CD49d that together with CD29 form the integrin α4β1 (VLA-4). Three days following transfection, cells were labeled with anti-CD49d and anti-CD49e monoclonal antibodies and analyzed by flow cytometry. A decrease on the surface expression of CD49e receptor was confirmed, but no significant differences in the cell surface expression of CD49d was observed (Figure 2A). Of note, we also analyzed the gene expression of integrin β1 subunit (CD29) by real-time quantitative PCR and there was no reduction of gene expression levels when compared scramble-siRNA *versus* CD49e-siRNA transfected cells (Figure 2B). Also, fibronectin expression, at both mRNA and protein levels, was not significantly changed (Figure 2C-D).

Conjointly, these data tell us that CD49e-siRNA specifically repress the expression of the corresponding gene, but does not interfere with the expression of its natural ligand, fibronectin, neither to the expression of the other integrin-type fibronectin receptor, VLA-4.

**Figure 3:**
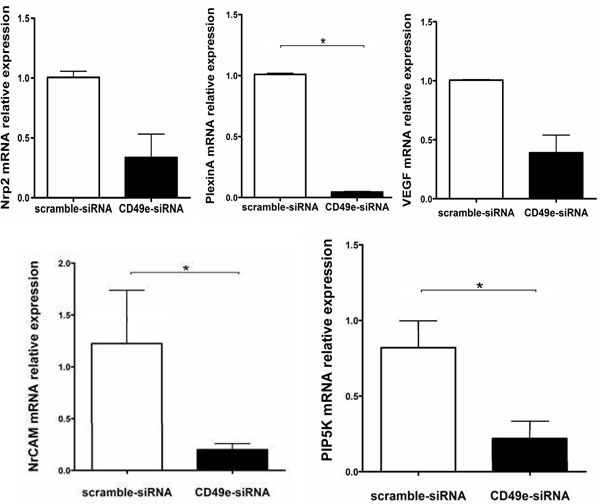
**siRNA-induced knowndown of CD49e in cultured human thymic epithelial down-regulates other genes related to cell adhesion.** The expression of neuropilin-2 (Nrp2), vascular endothelial growth factor (VEGF) and plexin A mRNA are seen in the upper panels, whereas neuronal cell adhesion molecule (NrCAM), and phosphatidylinositol-4-phosphate 5-kinase (PIP5K) are seen in bottom panels. In all cases, real-time quantitative PCR was applied to compare CD49e-siRNA (black bars) with scramble-siRNA (open bars) treated human TEC. Analyses were carried out 48 hours post-transfection. Results were normalized to β-glucuronidase mRNA and GAPDH mRNA and they are expressed relative to the mRNA levels in CD49e-siRNA versus scramble-siRNA in transfected cells; results are representative of three independent experiments performed in triplicate. Bars the mean values ± SE; and the data were validated using Mann-Whitney test (p ≤ 0.05).

Nevertheless, real-time quantitative PCR analysis revealed that members of other adhesion receptor gene families were modulated. This was the case for neuronal cell adhesion molecule, as well as neuropilin-2 and its corresponding signaling transducer plexin A1. Interestingly, gene expression of another natural neuropilin ligand, VEGF, was also down-regulated in CD49e-siRNA treated TEC, as compared to control scramble-siRNA treatment (Figure 3).

Finally, we found that gene expression of one member of the integrin signaling cascade, phosphatidylinositol-4-phosphate 5-kinase (PIP5K), was down-regulated in CD49e-siRNA treated TEC.

### Gene expression profile in CD49e RNA silenced human TEC

The above findings, together with previous data showing that blockade of CD49e with a specific antibody directly stimulated the production of both TGF-β (transforming growth factor-β) and PAI-1 (plasminogen activator inhibitor-1) in human mesangial cells [16], led us to further explore the effect of CD49e gene silencing in cultured TEC, using DNA microarray strategy. The relative abundance of each transcript was analyzed 48 hours after transfection. Three independent experiments were performed and the efficiency of CD49e depletion was confirmed by real-time quantitative PCR (Figure 1A). The microarray analysis identified 131 genes differentially expressed following knockdown of the CD49e gene, when compared with TEC cultures treated with scramble-siRNA. Among these, 65 genes were down-regulated and 66 genes were up-regulated (see each gene separately in Additional file 1 and 2).

**Figure 4:**
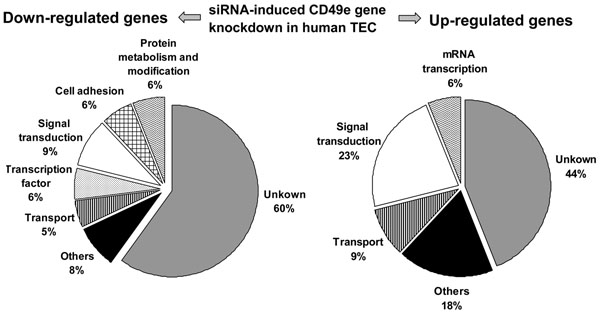
**Classification of genes differentially expressed by human thymic epithelial cells after treatment with CD49e-siRNA versus scramble-siRNA.** The figure shows that most of genes up- or down-regulated in cultured human TEC under treatment with CD49e-siRNA (as compared to scramble-siRNA) are not annotated. Yet, among the down-regulated genes with defined functions, 6% code for adhesion molecules. Additionally, a large number of genes related to signal transduction was modulated in CD49e silenced TEC: among those genes whose expression was down-regulated they represented 9%, and corresponded to 23% of the whole amount of up-regulated genes. The classification of 65 down and 66 up-regulated genes in silenced cells to biological functions is given in left and right panels, respectively. The classification into functional categories was defined according to the NCBI, SOURCE (http://smd.stanford.edu/cgi-bin/source/sourceSearch) and DAVID (http://david.abcc.ncifcrf.gov/) databases.

The differential expressed genes were classified in categories according to biological function (Figure 4). Among the 65 down-regulated genes, 6% corresponded to those reported above, concerning cell adhesion molecules. Genes related to signal transduction and transcription factors represented 9% and 6% of the down-regulated genes, whereas 6% were related to protein metabolism, and 5% to intracellular transport. Nevertheless, 60% of the down-regulated genes are still not annotated.

As shown in figure 4, among the 66 up-regulated genes, 44% are not yet annotated, and 23% belong to the functional category of signal transduction. Some of these genes, like phospholipase Cγ1 (PLCγ1), cysteine-rich PAK1 inhibitor (CRIPAK), V-raf murine sarcoma viral oncogene homolog B1 (B-raf) and SHC (Src homology 2 domain containing) transforming protein 1 (SHC1), are involved in the integrin signaling pathway, thus suggesting the existence of compensatory mechanisms taking place within the integrin-related circuitry.

### RNAi mediated loss of CD49e expression leads to a decrease in TEC/ Thymocyte adhesion

**Figure 5:**
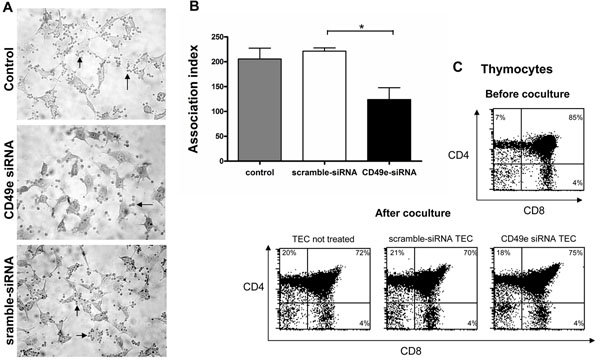
**Decrease of thymocyte adhesion to human thymic epithelial cells following siRNA-induced knockdown of the CD49e gene.** Panel **A** depicts representative microscopic fields of human TEC cultures were transfected with scramble-siRNA or CD49e-siRNA, or that remained untreated. Three days post-transfection thymocytes were led to adhere for one hour, being then fixed and were stained with Giemsa. Adhered thymocytes (seen as darkly stained spots, arrows) were more numerous in untransfected cells and scramble-siRNA transfected cells, when compared to CD49e-siRNA silenced cells. Original magnification X100. Such differences in the numbers of adhered thymocytes to TEC were quantified and data are shown in panel **B**, in which the adhesion degree is plotted as association index, in which values correspond to mean ± SE (see material & methods section). Differences were statistically evaluated by Student’s *t*-test. Data are representative of four separate experiments and were significanly different between scramble-siRNA transfected cells *versus* CD49e-siRNA silenced cells. *p<0.02. Panel **C** reveals that, the significant reduction in thymocyte adhesion comprises all CD4/CD8-defined thymocyte subsets, herein ascertained by cytofluorometry, since their proportions are similar in the three conditions. For comparison, we also included the typical CD4/CD8 profile of human thymocytes before co-culture. Lymphocyte gate was defined by side-scatter and forward scatter dot plots.

In a last set of experiments, we addressed the functional consequences of CD49e-siRNA-triggered down-regulation of CD49e expression in TEC, upon their ability to interact with developing thymocytes. For this purpose, we evaluated thymocyte adhesion in TEC transfected with CD49e-siRNA *versus* untreated or scramble-siRNA treated. Thus, CD49e-siRNA transfected cells had decreased number of adhered thymocytes compared to cells transfected with scramble-siRNA or untreated cells (Figure 5A-B).

It is noteworthy that, although a precise quantitative analysis was not done, we did not notice any clear-cut change in TEC growth, with cultures being rather similar among the various groups.

We then evaluated whether adhesion blockade targeted preferentially a given CD4/CD8-defined thymocyte subset (Figure 5C). This was not the case, since all immature and mature subsets were similarly affected after adhesion in CD49e-siRNA, when compared to scramble-siRNA or untreated cells.

Taken together, our observations confirm the importance of the fibronectin receptor VLA-5 in TEC/thymocyte adhesion, affecting interactions with both immature and mature thymocytes.

## Discussion

During T cell development, bone marrow-derived precursors migrate towards the thymus. Developing thymocytes travel within the cortex and medulla through cell-cell and cell-extracellular matrix contacts and also in response to soluble secretory moieties such as cytokines and chemokines [1,2]. During this process, there is a requirement of several time-dependent adhesion events between thymocytes and cells of the thymic microenvironment. Among the various molecular mechanisms involved in such heterocellular adhesion, one is represented by fibronectin/integrin mediated interactions. In particular, previous studies using anti-CD49e blocked antibody have shown that such an adhesion is partially under control of the FN/VLA-5 ligand/receptor pair [4,13-15].

Herein, we applied the RNA interference strategy to silencing the CD49e gene, thus disrupting the integrin VLA-5 in human thymic epithelial cells. Although being transient, in most experiments we got a high efficiency in abrogating the corresponding CD49e mRNA, as well as its translation into the α5 integrin subunit (as ascertained by its detection on the cell membrane). Moreover, the specificity of the siRNA knockdown of the CD49e gene was ascertained by the fact that no effect was seen with a scramble siRNA. Also, it is interesting that the expression of CD49d (α4 integrin subunit of the VLA-4 fibronectin receptor) quantified by a specific antibody for the CD49d molecule using flow cytometry, remained unchanged in CD49e-siRNA-treated TEC. One could argue that another CD49d-containing integrin, namely α4β7 integrin might be modulated by CD49e knock down. However, this is not the case, since the VLA-4 integrin (CD49d/CD29) is actually the only CD49d-containing integrin constitutively expressed by this human thymic epithelial cell preparation, as previously ascertained by immunoprecipitation and cytofluorometry [35]. In a second vein, although the expression of CD29 mRNA (the β1 subunit that forms the various members VLA integrin sub-family) also remain unchanged, we cannot discard the possibility that other members of the VLA family (for example the laminin receptor are modulated in this experimental conditions. Yet, in any case, from a conceptual point of view our findings unravel the independency of the regulation of genes coding for different ECM receptors.

It should be point out, however, other molecules related to cell adhesion/migration can be somewhat under control of CD49e. In this respect, we found a down-regulation of the gene coding for another adhesion receptor, namely neuronal cell adhesion molecule (NrCAM), which belongs to the L1 family of immunoglobulin-like cell adhesion molecules, initially discovered in nervous system [17]. In this regard, it is been described that NrCAM interacts with various integrins including α5β1, and that such interactions can support cell binding and migration [18].

NrCAM is part of a growing list of “neural” molecules, also including neuropilins, which are expressed in immune systems, including the thymus [see reviews] [19,20]. In addition to neuropilins, plexin A (that mediates neuropilin-induced intracellular signaling), and VEGF, one of neuropilin natural ligands have been described in the thymus [20]. In the human thymus, neuropilins mediate thymocyte migration [19,20]. All these genes are down-regulated following siRNA-induced CD49e knockdown in human TEC, strongly indicating the existence of a highly complex intracellular molecular circuitry in the thymic epithelium, controlling the expression of various genes involved in physiological guidance of developing thymocytes within the thymic lobules. It is conceivable that the mechanisms underlining such control may comprise direct as well as indirect effects of CD49e gene knockdown, as for example via modulation of specific microRNAs that target those genes, down- or up-regulated after CD49e silencing. Dissecting this circuitry shall provide a much better understanding of how the TEC network controls thymocyte migration and adhesion. In a second vein, the microarray analysis revealed that siRNA-induced knockdown of CD49e in cultured human TEC also deregulated the expression of signal transduction genes, some of them related with intracellular pathways triggered by integrins. The binding of fibronectin to α5β1 activates two major tyrosine kinase-dependent pathways, the focal adhesion kinase (FAK) pathway and the Shc pathway [21]. FAK is recruited to the ligand binding site and becomes activated through phosphorilation of tyrosine residues [22]. In turn, activation of FAK directly phosphorylates Src-family protein-tyrosine kinases [23]. Additionally, FAK can form an association with phosphatidylinositol 3-kinase (PI3K), and such an association activates PI3K and its signaling pathways [24], regulating, among other functions, the actin cytoskeleton [25]. The regulation of actin fibers by PI3K can be mediated by centaurin α-1, a phosphatidylinositol interacting protein, which directly interacts with F-actin, resulting in a decrease in stress fibers, as ascertained in Hela cells [26].

In our microarray data, we noticed that siRNA-induced CD49e gene silencing in human TEC resulted in up-regulation of the centaurin α-1 gene. Moreover, the down-regulation of phosphatidylinositol-4-phosphate 5-kinase type I (PIP5K) mRNA, that is the predominant kinase that synthesized phosphatidylinositol 4,5-bisphosphate [27,28] also indicates a decrease in stress fibers in TEC with CD49e knocked down. Although we have not directly tackled this issue, such a decrease in F-actin could explain, at least partially, the decrease in the ability of CD49e-siRNA-treated TEC to promote thymocyte adhesion.

Among genes down-regulated in human cultured TEC in which CD49e was knocked down by RNA interference, we also found the activin receptor-like kinase 1 gene (acvrl1), which encodes a type I cell-surface receptor (ALK1) for transforming growth factor-β1 (TGF-β1). This cytokine is involved in the control of T cell development through direct effect on both thymocyte and epithelial cell compartments [29]. Thus, it is conceivable that integrity of VLA-5 in human TEC is necessary to maintain proper levels one TGF-β receptor, thus allowing cross-talk between developing thymocytes and TEC.

Our results point some genes differential express due the CD49e silencing, however we can’t discard the effect of integrin crosstalk, a mechanism in which one integrin regulates the activation state of a different integrin in the same cell and this phenomenon involve the cytoskeleton associated protein talin [36]. In addition, the integrin crosstalk may be at the level of regulation the stability of integrin subunit mRNA [37]. We are just beginning to understand the molecular mechanisms that are involved in regulating the activation state of integrins and the molecular basis of crosstalk.

Finally, our data clearly show that knocking down the CD49e in human TEC, using RNA interference, results in a significant decrease in the ability of these cells to allow thymocyte adhesion. Such a decrease comprised all CD4/CD8-defined thymocyte subsets, a finding that is in accordance with the data showing that variable amounts of VLA-5 are expressed throughout the stages of intrathymic T cell differentiation [11]. Whether other integrin-mediated TEC/thymocyte adhesion interactions are also modulated after CD49e gene knockdown (for example the VLA-6/laminin binding) is presently under investigation.

## Conclusion

In conclusion, the present study shows that siRNA-mediated knockdown of the fibronectin receptor α5 integrin chain CD49e in human thymic epithelial cells modulates, directly or indirectly, the expression of multiple genes and decreases thymocyte adhesion. Among the genes differentially expressed other cell adhesion related molecules were down-regulated, including neuronal cell adhesion molecule, neuropilin-2, plexin A and vascular endothelial growth factor. Additionally, more than 130 genes are modulated in human TEC following knockdown of CD49e. Some of these genes are related to intracellular signaling pathways that can be triggered following fibronectin binding to VLA-5. Conceptually, these findings unravel the complexity of gene regulation, as regards integrin-integrin crosstalk and key genes involved in the heterocellular cell adhesion between developing thymocytes and the major component of the thymic microenvironment. At biological level, analyses of the effects result from manipulation the expression of an integrin subunit are complicated by the integrin crosstalk. Further characterization of the relationship between these genes and their corresponding proteins will offer a more complete understanding of the cellular pathways affected by CD49e gene knockdown and may lead to a better comprehension of the tightly regulated process in which different thymic microenvironmental cells, including TEC, support interactions and signals required for the proper intrathymic T cell development.

## Methods

### Thymus samples and thymic epithelial cell cultures

Surgical discarded thymic tissues were obtained from children undergoing cardiac surgery, aged from 1 day to 3 years. Experimental procedures with human thymic fragments have been approved by the Oswaldo Cruz Foundation Ethical Committee for Human Research.

The human TEC line was obtained from an infant thymus by an explant technique and limiting dilution cloning [12], being kindly provided by Dr. Maria Luiza Toribio (Universidad Autonoma de Madrid, Madrid, Spain). TEC cells were cultured in 10% fetal bovine serum-supplemented RPMI 1640 medium at 37°C in a 5% CO_2_ atmosphere.

### CD49e (α5 integrin subunit) gene silencing

We used two siRNAs conjugated with alexa-488 (*Qiagen, Hilden, Germany*): a) unrelated siRNA, bearing the same nucleotides, but scramble organized and not complementary to any gene in mammals, b) specific CD49e-siRNA, designed to specifically silence the expression of CD49e. Semi-confluent cultures of human TECs cell line were transiently transfected with 2.5 nM of CD49e-siRNA or scramble-siRNA using Hiperfect reagent (Qiagen) in 24 well plates, following manufacturer’s instructions.

After transfection, cells were cultured during 24 to 96 hours in RPMI medium as above mentioned. Transfection efficiency was confirmed by flow cytometry 24 hours after transfection. Gene knockdown was also confirmed by real-time quantitative PCR 48 hours after transfection and protein silencing was confirmed by immunohistochemistry and flow cytometry 72 hours after transfection.

### RNA Isolation and real-time quantitative PCR

Confluent TEC monolayers in 24 well plates untreated or transfected with control scramble-siRNA or CD49e-siRNA were dissociated in 1 ml of Trizol reagent (*Invitrogen Life Technologies, Carlsbad, CA, USA*) 48 hours after transfection, according to the manufacturer’s instructions. The isolated RNA was dissolved in diethyl-pyrocarbonate-treated water and quantified by UV spectrophotometry. We used only those DNA and phenol-free RNA preparations. The integrity of RNA samples was evaluated by conventional agarose gel electrophoresis.

Total RNA extracted from TEC was reverse transcribed by using the Superscrit II reverse Transcriptase kit (*Invitrogen*) following the manufacturer`s recommendations. cDNA was diluted 1:5 in sterile water and used for real-time quantitative PCR. Alternatively, the total RNA was reverse transcribed and Cy3/Cy5 labeled by using *CyScribe Post-Labelling* Kit (*GE Healthcare*) for cDNA microarrays hybridizations.

### Real-time quantitative PCR

The PCR primers were designed based on the sequences reported in NCBI GenBank (http://www.ncbi.nlm.nih.gov/pubmed/) with the Primer Premier 5 software (http://www.brothersoft.com/primer-premier-73311.html) (Additional file 3). The prepared cDNAs were amplified using Power SYBR Green Master Mix (*PE Applied Biosytems, Foster City, CA, USA*) in a 25μL reaction mixture in an ABI PRISM 7700 Sequence Detection System (*PE Applied Biosytems*). All standard dilutions were run in duplicate and the average value of gene expression was used. Standard curves were accepted when the slopes were between -3.74 and -3.32 (corresponding to PCR efficiencies of between 85 and 100%).

Primer pairs were evaluated for integrity by analysis of the amplification plot, dissociation curves, and efficiency of PCR amplification. For NrCAM, PIP5K and GAPDH genes we used taqman probes, primers and Universal Taqman Master Mix, all purchased from Applied Biosystems.

PCR conditions were 10 minutes at 95°C, followed by 40 cycles of 15 seconds at 95°C and 60°C for 1 minute. PCR amplification of the housekeeping gene encoding β-glucuronidase was performed during each run for each sample to allow normalization between samples. Relative quantification and calculation of the range of confidence was performed using the comparative δδCT method.

### cDNA microarray method

Gene expression of TEC transfected with control scramble-siRNA or CD49e-siRNA was assessed using glass slide cDNA microarrays, containing 4,500 sequences (in replicates) from the human expressed sequence tags (ESTs) cDNA library (IMAGE Consortium, http://image.hudsonalpha.org). The microarrays were prepared based on published protocols using 0.75-1.0 kb PCR product from the cDNA clones [30]. The microarray were constructed on silane-coated UltraGAPS slides (#40015, Corning ®, New York, NY, USA) using a Generation III array spotter (Amersham Biosciences Molecular Dynamics Sunnyvale, CA, USA) according to the manufacturer’s instructions, and cross-linked using an ultraviolet cross-linker. The cDNA complex probes were prepared by reverse transcription using 10 µg of total RNA from controls or CD49e silenced TECs which were labeled using CyScribe post-labeling kit (GE Healthcare), and oligo dT12-18 as a primer. The 15h period of hybridization followed by washing was performed in an automated slide processor (Amersham Biosciences) and microarrays were scanned in a Generation III laser scanner (Amersham Biosciences). cDNA complex probes from controls or CD49e silenced TECs were Cy5-labeled. Cy3-labeled pool, consisting of equimolar amounts of total RNA from different human cell lines (Jurkat, Hela, Hep-2 and U343), served as reference in the two-color hybridizations. Microarrays raw signal values as well as fold-change values for this entire data set are publicly available at the National Center for Biotechnology Information (NCBI) Gene Expression Omnibus (GEO) database, (GEO series accession no. GSE18116 at http://www.ncbi.nlm.nih.gov/geo/).

### Microarray statistical analysis

All analyses were performed using the freely available statistical software and graphics *R* environment [31]. The “SPOT” software package (http://www.experimental.act.cmis.csiro.au/Spot/index.php) was used to identify spots by adaptive segmentation method and subtract backgrounds utilizing morphological opening approach. Information thus recovered was analyzed using the Limma package [32]. All data were normalized to remove from the expression measures any systematic bias which arise due to the microarray technology itself, rather than differences between the probes or between RNA samples targets hybridized to the arrays. This was done by the print-tip Loess method which corrects for dye bias and intensity within each group of adjacent spots printed by one pin (print-tip). Spots that were too small or large, based on its area, were kept in the analysis but down-weighted. Differentially expressed genes were ranked on Bayesian posterior log odds calculated with Limma. The empirical Bayes method was used to shrink the gene-wise sample variances towards common values, thus augmenting the degrees of freedom for the individual variances. This approach combines expression ratios and their variability between replicates to rank the genes [33]. Statistical significance between groups of interest -- scramble-siRNA versus CD49e-siRNA -- was assessed for relevant contrasts using moderated *F* statistics as implemented in Limma package. In order to determine which genes were significantly modulated by CD49e-siRNA, we considered differentially expressed genes according to the following contrast of interest: CD49e-siRNA *versus* scramble-siRNA. We used Benjamini and Hochberg's [34] method to control the false discovery rate for the considered contrast.

### Immunofluorescence

Slides containing untreated TEC, or cells treated with CD49e-siRNA or corresponding scramble-control, were washed with PBS/1% BSA. Samples were incubated with anti-CD49e or anti-fibronectin (BD Biosystems, San Diego, USA) overnight at 8°C, and then incubated with secondary antibody for 30 minutes, using the anti-mouse Ig conjugated to phycoeritrin for revealing the anti-CD49e antibody and the rhodamine-couple anti-rabbit IgG for detecting the anti-fibronectin reagent. Immunostained samples were analyzed by confocal microscopy using the Zeiss LSM 510 or LSM 5 Pascal devices. Negative controls, in which primary antibodies were replaced by unrelated immunoglobulin was used alone, and did not generate any significant labeling.

### Cytofluorometry

Cytofluorometric analyses were performed using transfected an untransfected TEC cell in different periods of incubation, as well as adherent thymocytes. Following 24 hours of transfection TEC were washed and evaluated for siRNA alexa-488 staining and after 72 hours of transfection they were washed and staining with anti-CD49e or anti-CD49d monoclonal antibodies that specifically recognize the human corresponding molecules. Adherent thymocyte phenotyping was ascertained by direct cytofluorometry, using anti-CD49e/PE, anti-CD4/Percp and anti-CD8/APC monoclonal antibodies. All antibodies were purchased from Pharmigen/BD Biosystems). Cells were then evaluated by flow cytometer in a FACSCalibur (BD Biosystems) and analyzed using the CellQuest software (BD Biosystems).

### Thymocyte-Thymic epithelial cell adhesion assay

Influences of CD49e gene knockdown upon thymocyte interactions with thymic epithelial cells were tested using RNA interference for CD49e in human thymic epithelial cell line cultures. The ability of untreated or siRNA transfected TEC to bind thymocytes was evaluated by two distinct techniques. One approach was the co-culture in the 24 well plate for phenotyping analysis by flow cytometry of those thymocytes that remained adhered on the TEC monolayer. We exposed TEC monolayer to 50 thymocytes per TEC for one hour at 37°C in a 5% CO_2_ atmosphere, nonadherent thymocytes were gently washed out, and adhered thymocytes harvested and phenotyped. Cells were washed and subsequently submitted to three-color immunofluorescence staining as previously described [13]. A second approach to evaluate thymocyte/TEC adhesion was by direct counting, under the microscope, the numbers of thymocytes which had adhered to TEC cultures. TECs were trypsinized, replated to 8-well Labtek chambers (4 x 10^3^ cells/0.5 ml in each chamber) for 12-18h, exposed to 50 thymocytes per TEC for one hour at 37°C in a 5% CO_2_ atmosphere, nonadherent thymocytes were gently washed out, and coverglasses were fixed in cold absolute ethanol for five minutes and stained with Giemsa or used for immunoflurescence. Countings were integrated in the form of an association index (AI) and calculated using the following formula, previously validated for this type of analysis [13-15]:

At least 300 thymic epithelial cells with or without adhered thymocytes, were counted per well. These experiments were repeated at least three times, with two separate observers performed counting blind. We did not consider the growth rates, however, the evaluation by two different observers after transfections did not suggest any modifications in growth rates when compared no treated with treated cells.

## Abbreviations

acvrl1: activin receptor-like kinase gene; BSA: bovine serum albumin; DN: double negative cells (for the expression of CD4 and CD8); DP: double positive cells (for the expression of CD4 and CD8); SP: simple positive cells (for the expression of CD4 or CD8); ECM: extracellular matrix; FN: fibronectin; Nrp2: neuropilin-2; NrCAM: neuronal cell adhesion molecule; TEC: thymic epithelial cells; VEGF: vascular endothelial growth factor; PIP5K: Phosphatidylinositol-4-phosphate 5-kinase, type I;

## Competing interests

The authors declare that they have no competing interests.

## Authors` contributions

WS and LL conceived and designed the study, conducted the siRNA transfections, RNA extraction for expression profiling, performed the microarray assays, flow cytometry and immunohistochemistry assays and PCR validation, and wrote the manuscript. MRA carried out the analysis of microarray-related and statistical analyses, as well as the analysis of PCR. ACMN assisted in flow cytometry experiments and analyses. DAMC assisted in immunohistochemistry assays and respective analysis by confocal microscopy. DARO prepared the slides for microarrays and also performed the microarray experiments. MD conceived and designed some experiments, and participated in writing the manuscript. GAP and DAM designed the microarray experiments including expression analysis pipeline, GAP participated in writing the manuscript. WS conceived, designed and coordinated the study, and also wrote the manuscript. All authors read and approved the final version of manuscript.

## Supplementary Material

Additional file 1 Table I: Classification of the down- and up-regulated genes in siRNA-triggered CD49e silenced thymic epithelial cells. List of genes differentially express with annotation. Click here for file

Additional file 2 Tabel II: Down and upregulated genes in siRNA-triggered CD49e silenced thymic epithelial cells without annotation. List of genes differentially express without annotation. Click here for file

Additional file 3 Table III: Oligonucleotide sequences specifically tailored for used in real time PCR. GeneBank information and oligonucleotide sequences used in real time PCR. Click here for file
